# Regulation of p53 and Cancer Signaling by Heat Shock Protein 40/J-Domain Protein Family Members

**DOI:** 10.3390/ijms222413527

**Published:** 2021-12-16

**Authors:** Atsushi Kaida, Tomoo Iwakuma

**Affiliations:** 1 Department of Oral Radiation Oncology, Graduate School of Medical and Dental Sciences, Tokyo Medical and Dental University, Tokyo 113-8510, Japan; kai.mdth@tmd.ac.jp; 2 Department of Cancer Biology, University of Kansas Medical Center, Kansas City, KS 66160, USA; 3 Department of Pediatrics, Children’s Mercy Research Institute, Kansas City, MO 64108, USA

**Keywords:** HSP40, J-domain proteins, molecular chaperone, wild-type p53, mutant p53, tumor suppressor, cancer signaling

## Abstract

Heat shock proteins (HSPs) are molecular chaperones that assist diverse cellular activities including protein folding, intracellular transportation, assembly or disassembly of protein complexes, and stabilization or degradation of misfolded or aggregated proteins. HSP40, also known as J-domain proteins (JDPs), is the largest family with over fifty members and contains highly conserved J domains responsible for binding to HSP70 and stimulation of the ATPase activity as a co-chaperone. Tumor suppressor p53 (p53), the most frequently mutated gene in human cancers, is one of the proteins that functionally interact with HSP40/JDPs. The majority of p53 mutations are missense mutations, resulting in acquirement of unexpected oncogenic activities, referred to as gain of function (GOF), in addition to loss of the tumor suppressive function. Moreover, stability and levels of wild-type p53 (wtp53) and mutant p53 (mutp53) are crucial for their tumor suppressive and oncogenic activities, respectively. However, the regulatory mechanisms of wtp53 and mutp53 are not fully understood. Accumulating reports demonstrate regulation of wtp53 and mutp53 levels and/or activities by HSP40/JDPs. Here, we summarize updated knowledge related to the link of HSP40/JDPs with p53 and cancer signaling to improve our understanding of the regulation of tumor suppressive wtp53 and oncogenic mutp53 GOF activities.

## 1. Introduction

Tumor suppressor p53 (p53) is a transcription factor that regulates the expression of genes involved in cell cycle arrest and apoptosis, thereby functioning as a tumor suppressor [1,2]. Under non-stressed conditions, the level and activity of wild-type p53 (wtp53) are tightly regulated at a low level mainly through its degradation by the E3 ubiquitin ligase MDM2. Upon genotoxic stresses, p53 protein is post-translationally modified by phosphorylation and acetylation to be stabilized and transcriptionally activated, leading to cell cycle arrest, senescence, and DNA repair for cell survival or apoptosis for cell death [3,4,5,6,7]. While p53 protein mainly localizes to the nucleus, p53 is also detected in the cytoplasm, endoplasmic reticulum (ER), and mitochondria, thereby contributing to a variety of cellular activities [8,9,10,11,12]. Thus, wtp53 prevents cells from undergoing tumorigenesis and is hence called the guardian of the genome [2]. Mutations in the *p53* gene are one of the most frequent events in human cancers [13]. The majority of p53 mutations are missense mutations with single amino acid changes in the DNA binding domain, resulting in the production of mutant p53 (mutp53) proteins. Mutp53 is roughly classified into two types, class I and class II, according to the sites of mutations [14]. Class I is a DNA contact type, in which a mutation occurs in amino acids that directly bind to the p53-responsive elements in DNA to impair the p53’s sequence-specific DNA binding activity without robust changes in the protein structure. Class II is a structural or conformational type, in which a mutation results in a robust change in the p53 structure (misfolding), resulting in loss of the DNA binding activity, although not all mutants lose the DNA binding activity. Intriguingly, mutp53 not only loses wtp53’s tumor suppressive functions, but also promotes cancer progression, metastasis, and drug resistance independent of wtp53, referred to as gain of function (GOF) [14,15,16]. The GOF mechanisms are mainly caused by binding of missense mutp53 with tumor suppressors (e.g., p63, p73, MRN complex) to inhibit their functions or with oncoproteins (e.g., ETS2, SREBP2, VDR, NF-Y) to enhance their functions [17,18,19,20,21]. Increasing evidence indicates that accumulation of mutp53 protein is crucial for oncogenic GOF activities. Increased levels of mutp53 by MDM2 depletion and genotoxic stress enhance the GOF activities, including cancer metastasis [22]. While mutp53 is stabilized or degraded by similar mechanisms as wtp53, several reports suggest the presence of distinct mechanisms of mutp53 stabilization or degradation from those of wtp53 [23,24,25,26]. Thus, the exact mechanisms behind the stabilization or degradation of wtp53 and mutp53 are not fully understood.

Molecular chaperones play a central role in protein quality control, which involves multiple cellular processes including protein synthesis, folding, unfolding, stabilization, and degradation, thus maintaining cellular homeostasis and protecting cells from endogenous and environmental genotoxic stresses, such as heat shock, hypoxia, infection, chemicals, and radiation [27,28]. Exposure of cells to stress prompts molecular chaperones to capture protein intermediates, which promotes the protein folding or refolding to prevent them from misfolding and aggregation [25,29,30]. Of various types of molecular chaperones, heat shock proteins (HSPs) are ubiquitously expressed in cells and are highly conserved among all organisms. In mammals, HSPs are classified into six families, based on the molecular size, including small HSPs, HSP40, HSP60, HSP70, HSP90, and HSP100. HSP70 and HSP90 are involved in protein folding or refolding, as well as stabilization or degradation, leading to control of cell cycle, proliferation, and apoptosis [31,32,33,34,35]. The activity of HSP70 must be precisely controlled to exert a wide range of functions. While the basal ATPase activity of HSP70 is low, HSP40, which is generally considered as a co-chaperone of HSP70, interacts with HSP70 and stimulates ATP hydrolysis together with client proteins [36,37]. Thus, binding of HSP40 is important to drive the HSP70 functions.

**Figure 1:**
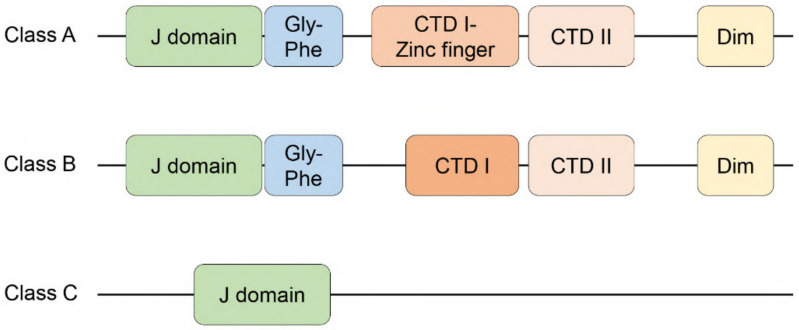
Structures of three types of HSP40/JDPs. Class A and B have the N-terminal J-domain, a Gly-Phe rich region (Gly-Phe), two C-terminal β-barrel domains (CTD I and CTD II), and a dimerization domain (Dim). A zinc-finger domain in the CTD I (CTD I-Zinc finger) is specific to the class A. Not all class B HSP40/JDPs contain CTD II and dimerization domains. J-domain containing proteins that do not fit to the criteria of class A or class B are classified as class C.

HSP40 is the largest family of the HSP families, consisting of over fifty members [37]. HSP40 is also known as J-domain proteins (JDPs) because it contains a highly conserved amino acid region, called “J domain”. Especially, the most conserved sequence, His–Pro–Asp (HPD) motif, is crucial for stimulating the ATPase activity of HSP70 [38]. HSP40/JDPs are categorized into three groups: Class A (DNAJA), class B (DNAJB), and class C (DNAJC) (Figure 1). Class A and B HSP40/JDPs are comprised of the N-terminal J-domain, a Gly-Phe rich region, two C-terminal β-barrel domains (CTD I and CTD II) containing substrate-binding regions, and a dimerization domain (Dim). A zinc-finger domain is further inserted into the CTD I in the class A HSP40/JDPs. Only a few of class B HSP40/JDPs (DNAJB1/HDJ1, DNAJB4/HLJ1, DNAJB5, DNAJB11/ERdj3/HEDJ) contain both the CTD II and dimerization domains, while other class B HSP40/JDPs lack either or both domains [37]. Any HSP40/JDPs that do not satisfy the criteria of either class A or B are classified into class C. There is little similarity in the sequence except for the J domain of HSP40/JDPs, which may explain the diverse functions of HSP40/JDPs and the exquisite regulation of HSP70 activity [37]. In addition to functioning as a co-chaperone of HSP70, several HSP40/JDPs (e.g., bacterially purified Ydj1: Yeast DNAJA1/HDJ2 ortholog [39,40], human DNAJB1/HDJ1 [41], human DNAJB6/MRJ [42], human DNAJB8 [42], RSP16: *C. reinhardtii* DNAJB13 ortholog [43], bacterially purified mouse DNAJC10/ERdj5 [44], Cwc23: Yeast DNAJC17 ortholog [45]) have some functions independent of HSP70. The HSP70 independency is shown using in vitro purified systems without HSP70, HSP70-lacking cell-based assays, or mutant JDPs that cannot bind to HSP70 or lack an active J domain with mutations in the HPD motif. Importantly, HSP40/JDPs are the first line of molecular chaperones that detect misfolded proteins and protein aggregates.

Increasing evidence indicates that HSP40/JDPs play roles in tumor suppression or progression, and some of them have been shown to regulate the levels and activities of wtp53 and mutp53. Intriguingly, the consequences of interactions between HSP40/JDPs and p53 are varied. A recent review article describes the effects of molecular chaperones on the wtp53 and mutp53 functions [25]. However, there is no review article summarizing the functional association of HSP40/JDPs with p53 and cancer signaling. Here, we focus on compiling reports related to the regulation of p53 activities and cancer progression by HSP40/JDPs, which may help design novel and appropriate targeted cancer therapies.

## 2. Effects of HSP40/JDPs on p53 Activity and Cancer Signaling

### 2.1. Class A HSP40/JDPs (DNAJA Proteins)

The class A HSP40/JDPs include DNAJA1, DNAJA2, DNAJA3, and DNAJA4 in mammals [37]. The roles of DNAJA1/HDJ2 and DNAJA3/Tid1 in cancer progression and regulation of p53 function are relatively well documented and are summarized below. DNAJA2 is implicated in cystic fibrosis and neurodegenerative diseases, mainly through degradation of misfolded cystic fibrosis transmembrane conductance regulator (CFTR) and inhibition of tau aggregation; however, its link to cancer development and p53 remains obscure [46,47,48]. Intriguingly, reduced mRNA expression of DNAJA4 by gene methylation is correlated with poor disease-free survival in stomach adenocarcinoma [49]. The hypermethylation status of *DNAJA4* is also observed in pediatric embryonal and alveolar rhabdomyosarcoma [50]. However, little is known about the association of DNAJA4 with p53.

#### 2.1.1. DNAJA1/HDJ2

DNAJA1, also known as HDJ2, is one of the best characterized HSP40/JDPs. DNAJA1/HDJ2 is implicated in a variety of diseases, including rheumatoid arthritis, oculopharyngeal muscular dystrophy, cystic fibrosis, and the neurodegenerative diseases [46,48,51,52,53,54]. This may be caused by DNAJA1’s abilities to interact with key proteins involved in these diseases.

- DNAJA1/HDJ2 and cancer

DNAJA1/HDJ2 is also implicated in progression of multiple types of cancer [55,56,57,58]. A study suggests the tumor suppressive activity, in which knockdown of DNAJA1/HDJ2 promotes spheroid formation, migration, and invasion of C6 rat glioblastoma cells and reduces survival of rats bearing C6 tumor xenografts [59]. However, most studies indicate the oncogenic role of DNAJA1/HDJ2. High DNAJA1/HDJ2 mRNA expression is associated with poor survival in patients with breast cancer, while DNAJA1/HDJ2 promotes an anti-apoptotic phenotype and invasiveness of pancreatic ductal adenocarcinoma cells [60,61]. DNAJA1/HDJ2 also promotes tumor growth and metastasis in human colorectal cancer (CRC) cell lines by interacting with and stabilizing cell division cycle 45 (CDC45) [56]. DNAJA1/HDJ2 also binds to an enzyme involved in extracellular matrix cross-linking and remodeling, transglutaminase 2 (TG2) that is associated with cell survival and cancer progression [62]. The anti-apoptotic activity of DNAJA1/HDJ2 is furthermore supported by a study showing that a DNAJA1-HSP70 complex inhibits nitric oxide-induced CHOP-mediated apoptosis, in which farnesylated DNAJA1/HDJ2 binds to BAX and inhibits the translocation of BAX to mitochondria [63]. However, whether p53 is involved in the aforementioned tumor suppressive or oncogenic functions of DNAJA1/HDJ2 has not been investigated.

- DNAJA1/HDJ2 and p53

Recent studies suggest that DNAJA1/HDJ2 stabilizes misfolded or conformational type of mutp53 to promote cancer progression (Table 1). Our group and others show that DNAJA1/HDJ2 binds to misfolded/conformational mutp53 to prevent CHIP ubiquitin ligase-mediated proteasomal degradation of mutp53 [23,55,64,65]. Indeed, DNAJA1/HDJ2 depletion reduces the levels of several misfolded/conformational mutp53 (R156P, V157F, R175H, C176F, Y220C, G245S), but not DNA contact mutp53 (R248L, R248Q) or wtp53 [23,55,65]. Intriguingly, mutp53 levels are not affected by HSC70 knockdown in CAL33 cells carrying p53^R175H^ [23], suggesting that stabilization of p53^R175H^ by DNAJA1/HDJ2 could be independent of HSC70, although further studies are required to clarify the dependency on the HSP70 family. Moreover, DNAJA1/HDJ2 promotes migratory and colony-forming potential of head and neck cancer cell lines expressing misfolded/conformational mutp53 (R175H, C176F), but not cells with DNA contact mutp53 (R248L, R248Q), wtp53, or p53-null [65]. Co-immunoprecipitation studies reveal specific binding of DNAJA1/HDJ2 with misfolded/conformational mutp53 (R175H, C176F), but not DNA contact mutp53 (R248L), which supports the misfolded/conformational mutp53-dependent function of DNAJA1/HDJ2 [65]. Intriguingly, prenylation of DNAJA1/HDJ2 is crucial for stabilizing the misfolded/conformational mutp53, since a C394S mutant DNAJA1/HDJ2 at the site of prenylation fails to rescue the reduced protein levels of p53^C176F^ in *DNAJA1/HDJ2*-knockout HN31 cells, unlike the full-length DNAJA1/HDJ2 [65], which is consistent with the previous observations [55,64,66]. 

DNAJA1/HDJ2’s transcription can be regulated by p53. An algorithm-based study predicts the presence of p53-responsive elements in the human *DNAJA1/HDJ2*’s promoter, which is confirmed by chromatin-immunoprecipitation studies using MCF7 cells (wtp53) [77]. Moreover, a recent report shows that wtp53 indirectly represses mRNA expression of DNAJA1/HDJ2 by inhibiting phosphorylation of heat-shock factor 1 (HSF1), the master regulator of the proteotoxic stress response [78]. However, DNAJA1/HDJ2 levels are unchanged following exogenous introduction of wtp53 in *mutp53*-knockout HN31 cells [65]. Thus, regulation of DNAJA1/HDJ2 expression by p53 may be dependent on the experimental setting or cellular context.

- DNAJA1/HDJ2 as a cancer therapeutic target

Given the cancer-promoting role of DNAJA1/HDJ2, DNAJA1/HDJ2 can be a potential target for cancer therapy. Moreover, DNAJA1/HDJ2 is shown to confer radio-resistance in human SF763 glioblastoma cells (p53^R158L^) [67]. By screening the NCI-approved oncology drugs collection in human chronic myelogenous leukemia HAP1 cell line with or without *DNAJA1/HDJ2* knockout, 41 compounds, including cabozantinib, clofarabine, and vinblastine, are identified as drugs that show synergy with *DNAJA1/HDJ2* loss [79]. While neither of these studies examines the mutp53 dependency of the radio- and chemotherapy-resistance, inhibition or depletion of DNAJA1/HDJ2 may increase the therapy efficacy. Since mutp53 increases radio- and chemotherapy-resistance [80,81,82,83], treatments of cancer cells carrying misfolded/conformational mutp53 with a DNAJA1/HDJ2 inhibitor may effectively increase the sensitivity to radio- and chemotherapies. Currently, no DNAJA1/HDJ2 inhibitor is clinically available, although 116-9e inhibits the DNAJA1/HDJ2 and HSP70 binding, and a chalcone compound, C86, appears to bind to and inhibit several HSP40/JDPs [84,85]. Identifying a compound that specifically inhibits DNAJA1/HDJ2 or multiple HSP40/JDPs would accelerate the development of targeted cancer therapy, specifically for cancers expressing misfolded/conformational mutp53.

#### 2.1.2. DNAJA3 (Tid1: Tumorous Imaginal Disc 1)

DNAJA3/Tid1 is mainly localized in the mitochondrial matrix to interact with mitochondrial HSP70 and their clients, and hence mitochondrial DNAJA3/Tid1 is responsible for maintaining mitochondrial DNA (mtDNA) integrity and mitochondrial membrane potential [86]. However, the splicing variants of DNAJA3/Tid1 (Tid1-S, Tid1-L) are localized in the cytosol [87]. While the exact functions of mitochondrial DNAJA3/Tid1 and the cytosolic variants remain to be elucidated, DNAJA3/Tid1 is involved in a variety of cellular processes, including proliferation, differentiation, senescence, survival, apoptosis, migration during embryonic development, skeletal muscle development, immunity, and viral infection [88,89,90,91,92,93,94,95,96]. Intriguingly, *DNAJA3/Tid1* knockout mice develop dilated cardiomyopathy with decreased copy number of mtDNA in cardiomyocytes [97].

- DNAJA3/Tid1 and cancer

DNAJA3/Tid1 is implicated in cancer development. In head and neck cancers, high DNAJA3/Tid1 protein levels are correlated with favorable outcome with reduced malignancy and recurrence by inhibiting the galectin-7-TCF3-MMP9 axis or inhibiting the activities of EGFR and AKT [88,98]. Moreover, immunohistochemistry studies using breast cancer tissues show that DNAJA3/Tid1 levels are inversely correlated with tumor malignancy and ErbB2 levels through direct interaction with ErbB2 to promote CHIP-mediated proteasomal degradation [99]. In lung adenocarcinoma, reduced DNAJA3/Tid1 protein levels are correlated with poor overall survival and increased EGFR levels [100]. A recent study in human hepatocellular carcinoma shows that reduced DNAJA3/Tid1 protein levels are associated with increased Nrf2 protein levels and colony-forming potential of human HCC cells, as well as poor clinical outcomes after surgery [101]. In human gastric cancer, reduced DNAJA3/Tid1 expression is correlated with a poor prognosis and increased lymph node invasion in patients. Indeed, knockdown of DNAJA3/Tid1 in gastric cancer cells increases cell migration and invasion with increased protein stability of galectin-7 [86]. Additionally, DNAJA3/Tid1 is shown to interact with von Hippel–Lindau (VHL) protein to induce degradation of HIF-1α, leading to inhibition of VEGF expression and angiogenesis [102]. These observations support tumor suppressive functions of DNAJA3/Tid1. On the other hand, there are a few reports suggesting the oncogenic function of DNAJA3/Tid1. In CRC, increased DNAJA3/Tid1 levels are correlated with colon cancer progression [103]. In non-small cell lung carcinoma (NSCLC), Tid1-S, but not Tid1-L, is required for the EGF-stimulated EGFR transportation into mitochondria, potentially leading to enhanced cancer cell migration and invasion. Indeed, high levels of Tid1-S and EGFR in the mitochondria are correlated with lymph node metastasis and poor overall survival of NSCLC patients [104]. Thus, the roles of DNAJA3/Tid1 in cancer suppression or progression appear to be dependent on the type of cancer and the presence of the variants. However, whether the tumor suppressive or oncogenic functions of DNAJA3/Tid1 are dependent on p53 remains unclear.

- DNAJA3/Tid1 and p53

p53 is another binding partner of DNAJA3/Tid1 (Table 1). Upon hypoxic and genotoxic stress, DNAJA3/Tid1 binds to p53 through the J domain, promoting p53 mitochondrial localization and transcription-independent apoptosis in multiple cancer cell lines [68,69]. Intriguingly, overexpression of DNAJA3/Tid1 enhances mitochondrial translocation of multiple mutp53 (R175H, L194F, R273H, E285K) in several breast cancer and glioblastoma cell lines, resulting in increased mitochondrial apoptosis regardless of the presence of hypoxic stress [68]. Thus, DNAJA3/Tid1’s binding to mutp53 may restore the transcription-independent mitochondrial apoptotic function of p53. Hence, increasing the DNAJA3/Tid1 levels or activity could be used as a strategy to induce apoptosis in p53-mutated cancers.

### 2.2. Class B HSP40/JDPs (DNAJB Proteins)

There are 14 subtypes of DNAJB proteins in mammals. DNAJB proteins have similar domains to DNAJA proteins except that they lack the zinc finger domain within CTD I, and not all DNAJB proteins contain CTD II and dimerization domains (Figure 1). Regulation of HSP70 activity by DNAJB proteins appears to be through intrinsic blockade of the J domain by the Gly-Phe rich region. This blockade can be released by the interaction of the CTD I region of DNAJB1/HDJ1 with the C-terminal EEVD tetrapeptide of HSP70, which triggers the binding of HSP70 to the J domain of DNAJB1 to activate HSP70’s ability to disaggregate amyloid fibrils [105]. Thus, DNAJB proteins exquisitely regulate HSP70 functions. DNAJB proteins are implicated in neuropathy (DNAJB2), muscular dystrophy or atrophy (DNAJB5, DNAJB6/MRJ), and primary ciliary dyskinesia (DNAJB13) [106,107,108,109,110,111,112,113,114]. Importantly, some DNAJB proteins, including DNAJB1/HDJ1, DNAJB4/HLJ1, DNAJB6/MRJ, DNAJB8, DNAJB9/MDG1/ERdj4, DNAJB11/ERdj3/HEDJ, and DNAJB12, are implicated in cancer progression [72,115,116,117,118,119,120,121,122,123,124,125,126,127]. Of these, DNAJB1/HDJ1 and DNAJB9/MDG1/ERdj4 are shown to bind to p53.

#### 2.2.1. DNAJB1/HDJ1

DNAJB1/HDJ1 is the best characterized DNAJB member. DNAJB1/HDJ1 is involved in neurodegenerative diseases, inflammation, and viral replication through binding to proteins involved in these diseases. These include aggregation-prone tau conformers, α-synuclein, melanoma differentiation-associated gene 5 (MDA5), and nucleoprotein component of influenza A virus ribonucleoproteins (RNPs) [128,129,130,131].

- DNAJB1/HDJ1 and cancer

DNAJB1/HDJ1 is also implicated in cancer progression and therapeutic resistance [24,132,133]. A fusion gene of *DNAJB1/HDJ1* and *PRKACA* (protein kinase cAMP-activated catalytic subunit alpha), resulting from an ~400 kb of in-frame deletion on chromosome 19, is found in nearly all cases of fibrolamellar hepatocellular carcinoma (FL-HCC) [115]. As a mechanism, the DNAJB1-PRKACA fusion protein accelerates the FL-HCC tumorigenesis by cooperating with the WNT pathway [133]. Moreover, DNAJB1/HDJ1 binds to mitogen-inducible gene-6 (MIG6), a tumor suppressor that inhibits the EGFR signaling, which decreases the protein level of MIG6 by enhancing its ubiquitination, leading to upregulation of the EGFR signaling pathway in A549 lung adenocarcinoma and HCT116 CRC cell lines [132,134]. Additionally, DNAJB1/HDJ1 is identified as a biomarker for cholangiocarcinoma [135]. Thus, DNAJB1/HDJ1 promotes the progression of multiple types of cancer, although it remains to be determined whether p53 is involved in these oncogenic activities of DNAJB1/HDJ1.

- DNAJB1/HDJ1 and p53

DNAJB1/HDJ1 binds to and regulates the activities of both wtp53 and mutp53 (Table 1). An in vitro study using purified proteins reveals that DNAJB1/HDJ1 forms a complex with p53 (both wtp53 and p53^R175H^) in the presence of HSC70 and ATP [136]. In support of this finding, Sugito et al. [137] observe the intracellular complex of p53^Y205C^, DNAJB1/HDJ1, and HSP70 in human oral squamous cell carcinoma HOC815 cells. DNAJB1/HDJ1 appears to contribute to tumor suppression via regulation of the wtp53 activity. Specifically, DNAJB1/HDJ1 binds to and stabilizes MDM2, a major ubiquitin ligase of p53; however, this interaction inhibits MDM2’s activity on p53, leading to p53 activation, while DNAJB1/HDJ1 knockdown increases cell proliferation and tumor growth of MCF7 cells in a manner dependent on wtp53 [41]. It should be noted that the C-terminal region of DNAJB1/HDJ1, lacking the J domain, is sufficient for interacting with and stabilizing MDM2, suggesting that DNAJB1-mediated stabilization of MDM2 is likely HSP70 independent. Silva et al. [138] also observe that DNAJB1/HDJ1, whose mRNA and protein levels are induced by trans-chalcone (TChal), binds to wtp53, leading to stabilization and activation of wtp53 in U2OS cells. These findings suggest the tumor suppressive role of DNAJB1/HDJ1.

However, Cui et al. [70] show that DNAJB1/HDJ1 binds to and induces degradation of Programmed Cell Death 5 (PDCD5), a positive regulator of p53-mediated apoptosis, in HCT116 cells, while knockdown of DNAJB1/HDJ1 enhances etoposide-mediated inhibition of colony formation with an increase in the PDCD5 levels and cell death in A549 cells. These results suggest that DNAJB1/HDJ1 inhibits wtp53’s apoptotic function through interaction with PDCD5 and contributes to cancer progression. Thus, the effects of DNAJB1/HDJ1 on the wtp53 activity are dependent on the cellular context or experimental settings.

Some studies have examined the functional interaction between DNAJB1/HDJ1 and oncogenic mutp53. Specifically, DNAJB1/HDJ1, together with HSP70, appears to facilitate binding of conformational mutp53 (R175H) with TAp73, a p53 family member. This complex induces chemoresistance to several DNA damaging reagents. Intriguingly, when MDM2 is overexpressed in cells carrying mutp53, this complex is inhibited by MDM2, which switches to the formation of mutp53-TAp73-MDM2 complex, leading to furthermore enhanced resistance to cisplatin, etoposide, and doxorubicin in SkBr3 and H1299 cells expressing p53^R175H^ [24]. This could explain why breast cancer patients with p53 mutations and high levels of MDM2 show poorer overall survival than those with p53 mutations or MDM2 overexpression alone [24]. The observation that DNAJB1/HDJ1 supports mutp53’s oncogenic GOF is also supported by a report by Parrales et al. [23] in which DNAJB1/HDJ1 contributes to the accumulation of p53^R175H^.

Intriguingly, Hiraki et al. [71] identify a natural compound, chetomin (CTM) as a compound that enhances the interaction of DNAJB1/HDJ1 with p53^R175H^ to restore the wtp53-like activity. CTM inhibits cell proliferation and tumor growth specifically in TOV-112D and CAL33 cells carrying p53^R175H^ with mRNA upregulation of p53 target genes, *p21*, *PUMA*, and *MDM2*, and increased DNA binding activity of p53^R175H^ to p53-responsible elements in these genes. Thus, the interaction of DNAJB1/HDJ1 with p53^R175H^ induced by CTM may promote refolding of p53^R175H^, rather than enhancing mutp53 GOF activity or stability as observed by other reports [23,24]. Since these experiments are not tested using cells lacking DNAJB1/HDJ1, it remains unclear whether the biological activity and mutp53 reactivation by CTM are solely dependent on DNAJB1/HDJ1. The discrepancies may be caused by the unappreciated function of CTM or differences in the cellular context. Together, whether DNAJB1/HDJ1 functions as a tumor suppressor or an oncogene appears to be dependent on the presence of wtp53 or mutp53, experimental settings, and the cellular context. 

#### 2.2.2. DNAJB9/MDG1/ERdj4

DNAJB9/MDG1/ERdj4 localizes to the ER and is upregulated by ER stress [139]. DNAJB9/MDG1/ERdj4 binds to and co-localizes with 78-kDa glucose-regulated protein (GRP78)/binding immunoglobulin protein (BiP), an ER chaperone in the HSP70 family member [139,140]. DNAJB9/MDG1/ERdj4 protects cells from ER stress and inhibits cell death, likely through stimulating the GRP78/BiP activity and stabilizing the GRP78/BiP’s binding to unfolded substrate proteins for protein folding. DNAJB9/MDG1/ERdj4 is also involved in the ER-associated degradation (ERAD) system by interacting with some ERAD substrates (misfolded surfactant protein C: SP-C, CFTR) and promotes their degradation [141,142]. Intriguingly, DNAJB9/MDG1/ERdj4 in the ER lumen promotes ERAD of a lipogenic transcription factor SREBP1c to inhibit lipogenesis, while DNAJB9/MDG1/ERdj4 in the ER membrane promotes the mTORC2 complex assembly in the cytosol to stimulate protein and ATP synthesis [143]. Indeed, overexpression of DNAJB9/MDG1/ERdj4 in the liver improves insulin sensitivity, restores protein synthesis, and reduces hepatic steatosis and adiposity in obese mouse models [143].

- DNAJB9/MDG1/ERdj41 and cancer

DNAJB9/MDG1/ERdj4 also plays a role in tumor suppression. In breast cancers, the DNAJB9/MDG1/ERdj4 mRNA level is lower than that in normal breast tissues, which is correlated with poor clinical outcomes [144]. Moreover, DNAJB9/MDG1/ERdj4 binds to and stabilizes F box/SPRY domain-containing protein 1 (FBXO45) to promote FBXO45-mediated ubiquitination and degradation of zinc finger E-Box binding homeobox 1 (ZEB1), leading to inhibition of migratory, invasion, and in vivo metastases of MDA-MB-231 cells [144]. However, involvement of p53 in these phenotypes has not been investigated.

- DNAJB9/MDG1/ERdj41 and p53

DNAJB9/MDG1/ERdj4 also interacts with wtp53 (Table 1). Lee et al. [72] show that DNAJB9/MDG1/ERdj4, whose mRNA expression is indirectly induced by wtp53, binds to wtp53 and inhibits p53-mediated apoptosis under genotoxic stress. While a DNAJB9/MDG1/ERdj4 mutant lacking the J domain cannot bind to wtp53, whether HSP70 is involved in the observed p53 inhibition by DNAJB9/MDG1/ERdj4 needs to be clarified. The same group also shows that DNAJB9/MDG1/ERdj4 overexpression inhibits the H-RAS^V12^-induced p53-dependent senescence in MEFs and promotes the cellular transformation [73]. In support of these findings, in human non-gestational choriocarcinoma samples, two missense mutations in the *DNAJB9/MDG1/ERdj4* gene are found (F46Y, H47R), while the introduction of site-specific mutations in the *DNAJB9/MDG1/ERdj4* gene in gestational choriocarcinoma JEG-3 cells results in reduced DNAJB9/MDG1/ERdj4 mRNA and protein levels with increase in the wtp53 levels [74]. These studies suggest the tumor-promoting function of DNAJB9/MDG1/ERdj4 by inhibiting wtp53, which is distinct from the aforementioned tumor-suppressive function of DNAJB9/MDG1/ERdj4 in breast cancer by Kim et al. [144]. Thus, the roles of DNAJB9/MDG1/ERdj4 in cancer suppression or progression could be dependent on the presence of wtp53 in cells or other cellular contexts.

#### 2.2.3. Other DNAJB Members and Cancer

- DNAJB4/HLJ1

DNAJB4/HLJ1 is not only implicated in myocardial infarction and Alzheimer’s disease [145,146], but also regulates cancer progression. In NSCLC and CRC, DNAJB4/HLJ1 levels are inversely correlated with clinical outcomes [147,148]. In invasive breast carcinoma, the DNAJB4/HLJ1 level is significantly lower, as compared to normal breast tissues, benign neoplasm, and ductal carcinoma in situ [149]. Tsai et al. [147] also show that exogenous expression of DNAJB4/HLJ1 reduces invasion, migration, proliferation, colony formation, and primary tumor growth of lung adenocarcinoma cells with increasing p21 levels; however, these phenotypes are p53-independent [147]. Overall, DNAJB4/HLJ1 functions as a tumor suppressor, but its functional relationship with p53 remains unclear.

- DNAJB6/MRJ

DNAJB6/MRJ is another DNAJB member implicated in cancers. High levels of DNAJB6/MRJ are associated with poor outcomes in patients with CRC, while knockdown of DNAJB6/MRJ in HCT116 and SW480 CRC cells inhibits invasion and pulmonary metastases with reduced IQ Motif Containing GTPase Activating Protein 1 (IQGAP1) levels, a scaffold protein of the MAP kinase pathway [123]. Lin et al. [150] also show that DNAJB6/MRJ promotes cell adhesion, migration, and invasion through stabilizing uPAR, as well as phosphorylation of FAK, ERK1/2, and AKT, in HCT116 cells. These reports suggest oncogenic roles of DNAJB6/MRJ. However, in breast cancer cells, DNAJB6/MRJ inhibits cancer progression by binding to HSPA8 and inhibiting Wnt/β-catenin signaling and epithelial-mesenchymal transition (EMT) [120,151,152]. Moreover, in esophageal squamous cell carcinoma (ESCC), nuclear localization of DNAJB6/MRJ is associated with favorable outcomes in patients with ESCC, while DNAJB6a, an isoform that contains a nuclear localization signal, reduces proliferation and xenograft tumor growth with reduced AKT signaling [122]. Thus, the function of DNAJB6/MRJ may be dependent on tissue type or the presence of the isoforms. Whether DNAJB6/MRJ has any impact on p53 activity needs to be determined as a future study.

- DNAJB8

DNAJB8 functions to protect against protein toxicity associated with polyQ aggregation diseases [153,154]. DNAJB8 is also identified as a factor that induces cancer stem-like properties, such as tumor-initiating ability and drug resistance in human CRC and renal cell carcinoma (RCC) cell lines [124,155,156]. However, the underlying mechanisms and the potential involvement of p53 remain to be elucidated.

- DNAJB11/ERdj3/HEDJ

DNAJB11/ERdj3/HEDJ is localized in ER and is associated with glomerular disease, autosomal-dominant polycystic kidney disease, and Gaucher’s disease [157,158,159,160]. DNAJB11/ERdj3/HEDJ is also upregulated in oral squamous cell carcinoma and hepatocellular carcinoma tissues [126,161]. Intriguingly, DNAJB11/ERdj3/HEDJ, together with HSP90, forms a complex with K1, a transmembrane glycoprotein encoded in the Kaposi sarcoma-associated herpesvirus (KSHV) genome, which is critical for the anti-apoptotic function of K1 in Kaposi sarcoma and other KSHV-related diseases [162]. It remains unclear whether the anti-apoptotic function of DNAJB11/ERdj3/HEDJ could be due to inhibition of p53 activity.

- DNAJB12

DNAJB12 is also an ER-associated HSP40/JDP [163]. ER stress induces degradation of DNAJB12 by the ubiquitin-proteasome pathway via ERAD complexes containing homocysteine-inducible ER protein (HERP), a suppressor/enhancer of Lin-12-like (Sel1L), and glycoprotein 78 (gp78) [127]. Intriguingly, DNAJB12 complexes with gp78 and Bcl-2 related ovarian killer (BOK), an ER-associated BCL-2 family member that regulates ER stress-induced apoptosis [127]. Depletion of DNAJB12 in Huh-7 hepatocellular carcinoma cells enhances apoptosis induced by proteotoxic agents and a proapoptotic chemotherapeutic agent (LCL-161), with an accumulation of BOK and caspase activation. There is no report showing any link between DNAJB12 and p53 [127].

### 2.3. Class C HSP40/JDPs (DNAJC Proteins)

Any HSP40/JDPs, which do not belong to either class A or B, are categorized into class C. The class C HSP40/JDPs are comprised of at least 32 members with the greatest diversity in their molecular sizes, structures, and functions [37,164]. While DNAJC proteins are implicated in a variety of diseases, limited studies have shown their roles in cancer progression [165,166]. Of 32 DNAJC members, DNAJC12, DNAJC15/MCJ, and DNAJC25 are implicated in cancer progression, while members linked to p53 include DNAJC2/ZRF1, DNAJC7/TPR2, and DNAJC9.

#### 2.3.1. DNAJC2/ZRF1

DNAJC2, also known as ZRF1 (Zuotin-Related Factor 1), is identified as a human ribosome-associated JDP that forms a complex with HSP70 and has been characterized as an epigenetic gene transcription regulator of stemness, development, and differentiation [167,168]. Mechanistically, DNAJC2/ZRF1 binds to ubiquitinated histone H2A and displaces the polycomb-repressive complex 1 (PRC1) from chromatin, leading to transcriptional activation [169,170].

- DNAJC2/ZRF1 and cancer

DNAJC2/ZRF1 is shown to enhance H-Ras-induced cellular senescence of MEFs by interacting with the *INK4/ARF* locus and upregulating p16^INK4a^ mRNA expression, thus displaying the tumor suppressive function. Knockdown of DNAJC2/ZRF1, indeed, enhances H-Ras-mediated transformation of MEFs [171]. 

On the other hand, DNAJC2/ZRF1 is overexpressed in human acute myelocytic leukemia (AML), and depletion of DNAJC2/ZRF1 results in decreased cell proliferation with increased apoptosis and cell differentiation, thus showing oncogenic function [172]. This may be caused, at least partially, by its binding to retinoic acid receptor α (RARα), since DNAJC2/ZRF1 knockdown enhances RA-mediated suppression of HL60 xenografts [172]. DNAJC2/ZRF1 protein levels are also upregulated in gastric cancer tissues as compared to non-tumor tissues, which is correlated with poor overall outcomes [75].

- DNAJC2/ZRF1 and p53

DNAJC2/ZRF1’s oncogenic function may be dependent on p53. Knockdown of DNAJC2/ZRF1 in human gastric cancer cell lines inhibits cell proliferation and migration and induces apoptosis with increased p21 levels, especially when cells carry wtp53 (Table 1). These phenotypes are minimally observed in cancer cells with p53 mutations and p53 null [75], suggesting that DNAJC2/ZRF1 inhibits the function of wtp53 to promote cancer progression. Thus, DNAJC2/ZRF1 acts as either a tumor suppressor or an oncogene, depending on the cellular context, including the cancer type and/or the presence of wtp53 in cells. 

#### 2.3.2. DNAJC7/TPR2

DNAJC7, also known as Tetratricopeptide Repeat 2 (TPR2), contains two TPR domains that bind to both HSP70 and HSP90, in addition to the J domain for stimulating ATP hydrolysis and polypeptide binding by HSP70 [173]. Accumulating studies suggest the involvement of DNAJC7/TPR2 in amyotrophic lateral sclerosis (ALS), likely through binding to natively folded tau and inhibiting tau aggregation [165,174,175]. DNAJC7/TPR2 binds to many other proteins, together with HSP70, including progesterone receptor, RAD9, a U-box E3 ubiquitin ligase UFD2a, and constitutive active androstane receptor (CAR)/Nuclear Receptor Subfamily 1 Group I Member 3 (NR1I3) [176,177,178,179]. However, the biological consequence of these interactions remains unclear.

- DNAJC7/TPR2 and cancer

DNAJC7/TPR2 is also implicated in cancer progression. Increased polyglutamylated-DNAJC7/TPR2 levels in sera may serve as a potential biomarker for early detection of RCC and are also associated with advanced stage and grade of RCC [180]. However, the underlying mechanism remains unclear.

- DNAJC7/TPR2 and p53

Kubo et al. [76] identify DNAJC7/TPR2 as a regulator of p53. DNAJC7/TPR2 binds to the DNA binding domain of p53. This interaction stabilizes and activates p53, leading to inhibition of the colony-forming potential of H1299 cells exogenously expressing wtp53, with increased p21, BAX, and MDM2 mRNA levels. Future studies are required to determine whether the tumor suppressive function of DNAJC7/TPR2 is entirely dependent on p53 or if DNAJC7/TPR2 could show any oncogenic functions in different experimental settings or cellular contexts. 

#### 2.3.3. DNAJC9

DNAJC9 activates the HSP70’s ATPase activity through the J domain as other HSP40/JDPs. DNAJC9 mRNA and protein levels are upregulated by various stress and mitogenic stimuli [181]. DNAJC9 mainly localizes to the nucleus; however, upon heat shock stress, DNAJC9 is exported to the cytoplasm and plasma membrane [181]. Recently, Hammond et al. [182] demonstrate that DNAJC9 forms a complex with a DNA replication licensing factor MCM2 and a histone H3-H4 dimer to recruit multiple HSP70 enzymes and fold histone H3-H4 dimers, for maintenance of a proper supply of histones during active replication and transcription. Intriguingly, DNAJC9 is implicated in familial recurrent corneal erosion dystrophy, epithelial recurrent erosion dystrophy, and schizophrenia [183,184].

- DNAJC9 and cancer

DNAJC9 is also implicated in cancer progression. DNAJC9 is upregulated in basal, HER2, and luminal B breast cancers, as well as in node-positive cervical squamous cell carcinoma [185]. By comprehensive analysis of transcriptomic profiles of HSP family genes in 9018 patients with 28 cancers, Liu et al. [186] show that DNAJC9 mRNA is upregulated in multiple different cancer types with prognostic values. These include adrenocortical carcinoma, AML, prostate adenocarcinoma, and lung adenocarcinoma. However, how DNAJC9 contributes to cancer progression remains to be elucidated.

- DNAJC9 and p53

While there is no report showing the regulation of p53 activity by DNAJC9, DNAJC9 appears to be a downstream target of p53 in the zebrafish system; an in silico screening of the zebrafish genome identifies a p53-responsive element in the zebrafish *DNAJC9* gene [187]. The chromatin-immunoprecipitation and luciferase reporter assays further confirm the interaction of p53 with the promoter region of the zebrafish *DNAJC9* gene.

#### 2.3.4. Other DNAJC Members and Cancer

- DNAJC12

DNAJC12 is implicated in dystonia and intellectual disability [188], mild hyperphenylalaninemia [188,189,190], and Parkinson disease in the Chinese Han population [191]. Some oncogenic roles of DNAJC12 have also been reported. In rectal cancer, increased DNAJC12 levels are correlated with poor chemotherapy response [192]. In gastric cancer, increased mRNA expression of DNAJC12 is correlated with cancer invasion, lymph node metastasis, and disease progression, thus having higher morbidity and mortality rates [193]. Moreover, in lung cancer, DNAJC12 levels are upregulated, while DNAJC12 knockdown reduces the malignant properties and tumor growth of lung cancer cells in vitro and in vivo by inhibiting activation of β-catenin [194]. However, none of these studies address if there is a link with p53.

- DNAJC15/MCJ

DNAJC15/MCJ is a unique member that localizes at the mitochondrial inner membrane. DNAJC15/MCJ functions as a negative regulator of the respiratory chain and inhibits the complex I activity and mitochondrial membrane potential, promoting ROS overproduction and ATP depletion [195]. DNAJC15/MCJ potentially acts as a tumor suppressor by promoting the release of pro-apoptotic molecules through the mitochondrial permeability transition pore complex [196]. Indeed, DNAJC15/MCJ is frequently hyper-methylated in multiple types of human cancer, including malignant pediatric tumors, neuroblastoma, Wilm’s tumor, melanoma, and breast cancer [197,198,199,200]. Reduced expression of DNAJC15/MCJ is also correlated with increased drug resistance, as well as increased levels of c-JUN protein and its downstream target ATP binding cassette subfamily B member 1 (ABCB1)/multiple drug resistance 1 (MDR1), in ovarian and breast cancers [200,201,202,203]. Whether p53 is involved in the tumor suppressive function of DNAJC15/MCJ has not been investigated.

- DNAJC25

DNAJC25 is relatively a new member of the HSP40/JDP subfamily C, and hence little is known about the function of this protein. In liver cancers, DNAJC25 mRNA expression is markedly reduced, while its overexpression induces apoptosis and inhibits colony formation of liver cancer cells [204]. In silico analyses suggest that DNAJC25 mRNA expression is also reduced in breast cancer tissues, and high DNAJC25 mRNA expression is correlated with favorable post-progression survival in breast cancer [205]. These results suggest the tumor suppressive role of DNAJC25, but the functional association of DNAJC25 with p53 remains to be elucidated.

## 3. Discussion

Both wtp53 and mutp53 proteins can be misfolded under a variety of cellular conditions (e.g., heat shock, genotoxic stress) like other proteins. These misfolded proteins as well as properly folded native proteins are detected by molecular chaperone systems, including HSPs, to be refolded, stabilized, or degraded. Indeed, both wtp53 and mutp53 are bound to and functionally regulated by the HSP system [206,207,208,209,210,211]. Since the HSP system has a great impact on the protein levels and functions, understanding the mechanisms by which HSPs detect and regulate the structure and functions of wtp53 and mutp53 would help design efficient p53-targeted anti-cancer therapies. At least fifty members of HSP40/JDPs are present in cells, and each has different clients [37]. This may explain the diverse regulation of target proteins including p53 by HSP40/JDPs and different biological outcomes. Several literatures suggest HSP70-independent functions of HSP40/JDPs in in vitro and in vivo [39,40,42,43,44,45]. Hence, whether the oncogenic or tumor suppressive functions of each HSP40/JDP are HSP70-dependent or -independent needs to be clarified in the future.

Recent studies suggest that wtp53 acts like mutp53 under specific conditions, referred to as pseudo-mutant p53. Indeed, previous studies indicate that wtp53 could be misfolded and form a mutant conformation, especially in the absence of specific chaperones, leading to reduced wtp53 transcriptional activity and cancer progression [212,213,214]. To support this finding, Arandkar et al. [215] observe that wtp53 in cancer-associated fibroblasts (CAFs), but not in normal fibroblasts, acquires misfolded conformation and rather enhances migration, invasion, and tumor growth of lung cancer cells. Moreover, a small subset of breast cancers with wtp53 display gene expression patterns similar to those carrying mutp53, including upregulation of genes specifically regulated by mutp53 (e.g., *PSAT1*, *TAP1*, *AurkA*, *CDC45*, *MAD2L1*, *ATL3*) [216]. Additionally, in a subpopulation of pre-leukemic hematopoietic stem/progenitor cells from primary human AML that carry DNA (cytosine-5)-methyltransferase 3 alpha (DNMT3A) mutations, high levels of pseudo-mutant p53 are dominantly detected over the wild-type conformation, while p53 in leukemic blasts shows mainly the wtp53 conformation [217]. It remains unknown how and under which conditions wtp53 conformation converts to mutant conformation and whether the process is reversible. Given the role of HSP40s/JDPs in protein folding and refolding, some members of HSP40s/JDPs could be involved in this process. 

Mutp53 can be reactivated by heat shock, second-site mutations, and chemical compounds, leading to restoration of the wtp53 activity. Specifically, temperature-sensitive mutp53 (e.g., A135V, V143A, G245S, R249S), which mainly localizes to the cytoplasm, relocates to the nucleus to be reactivated upon lowering temperature. Akakura et al. [218] show that HSC70 is associated with p53^A135V^ at 37 °C, but not at 32 °C, while the HSC70-containing complex masks the nuclear localization signal of mutp53 at 37 °C to prevent mutp53 from nuclear translocation. Moreover, a second-site mutation (e.g., H168R) reverts some of the chemical shift changes caused by the original mutation (e.g., R249S), leading to restoration of wtp53 conformation and the DNA binding activity [219]. Additionally, several chemical compounds have been identified to restore the wtp53 activity from mutp53. These include PRIMA-1MET, CTM, and SLMP53-2 [71,220,221,222]. PRIMA-1MET, that induces mutp53 reactivation and mutp53-dependent apoptosis, promotes nucleolar localization of mutp53, together with promyelocytic leukemia protein (PML) nuclear body-associated proteins (PML, cAMP responsive binding protein: CBP, HSP70), which may play a role in mutp53 reactivation [221,222]. As mentioned in the DNAJB1/HDJ1 section, CTM binds to DNAJB1/HDJ1 to reactivate p53^R175H^ [71]. SLMP53-2 is identified as a compound that inhibits viable cell proliferation of H1299 cells exogenously expressing mutp53 (R175H, Y220C, G245S, R280K) [220]. This compound restores wt-like conformation of mutp53 (Y220C) by enhancing the binding with HSP70, leading to upregulation of p53’s downstream target gene expression [220]. By acting as a co-chaperone of HSP70 and/or detecting misfolded protein structure, some HSP40/JDP members could regulate mutp53 reactivation. 

Understanding the mechanism of p53 regulation by HSP40/JDPs is crucial for developing novel p53-targeted therapies, given that current wtp53- and mutp53-targeting therapies have not yet been successful. Increasing the wtp53 activity specifically in tumors is required for successful targeted cancer therapy. For mutp53-expressing cancers, depletion or reactivation of mutp53 should be achieved without activating wtp53 in non-tumor cells. Given that HSP40/JDPs play central roles in the stabilization or degradation of wtp53 and mutp53, as well as reactivation of mutp53, controlling the p53 (wtp53, mutp53) levels and activity via HSP40/JDPs could be an alternative strategy for cancer therapy, instead of directly targeting p53.

## 4. Conclusions

**Figure 2:**
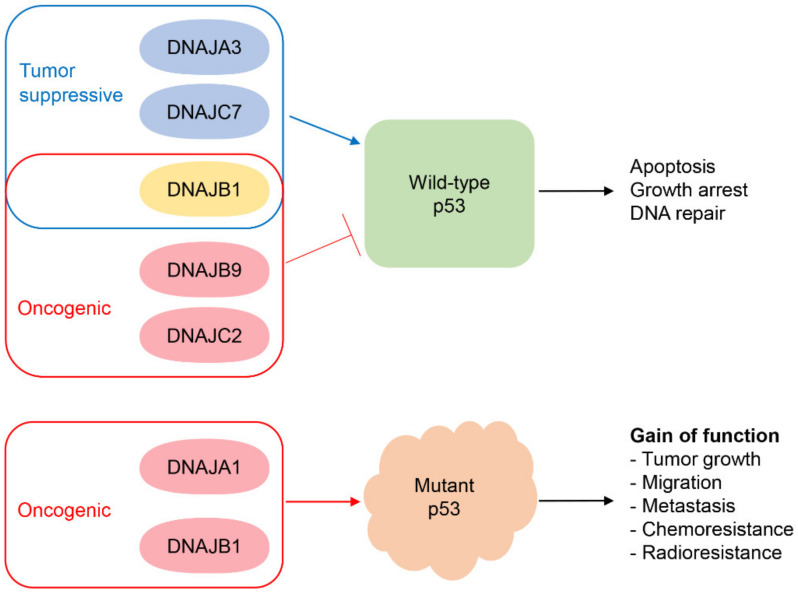
Regulation of wtp53 and mutp53 functions by HSP40/JDPs. DNAJA3 and DNAJC7 cooperate with wtp53 to enhance the tumor suppressive activity, while DNAJB9 and DNAJC2 have tumor-promoting functions by inhibiting the wtp53 activity. DNAJB1 can inhibit or enhance the wtp53 activity, likely depending on the cellular context and/or cancer type. DNAJA1 and DNAJB1 interact with mutp53 and contribute to the levels and activity of GOF mutp53.

We have given an overview of HSP40/JDPs mainly by focusing on their roles in cancer signaling and p53 functions (Figure 2 and Table 1). HSP40/JDPs detect misfolded structures of proteins and alter their localization, stability, or functions, therefore involved in numerous diseases including cancers. The activities of wtp53 and mutp53 can be fine-tuned by multiple HSP40s/JDPs, partially explaining diverse p53 functions in various cellular contexts and tumor microenvironments. Until now, only six HSP40/JDPs (DNAJA1, A3, B1, B9, C2, C7) have been found to regulate wtp53 and/or mutp53 activities, though 16 members are implicated in cancer progression. Identifying more HSP40/JDPs involved in cancer progression and p53 (wtp53, mutp53) activities, as well as the underlying mechanisms, will advance our knowledge of cancer progression and may accelerate the development of novel anti-cancer therapies.

## Figures and Tables

**Table 1:** Functional interactions between HSP40/JDPs and p53.

HSP40/JDPs	Type of p53 Proteins Interacting with HSP40/JDPs	Effects on p53	Dependency of HSP70 (UD: Undetermined)	References
DNAJA1/HDJ2	p53^R156P^, p53^V157F^, p53^R175H^, p53^C176F^, p53^Y220C^	DNAJA1/HDJ2 promotes oncogenic functions (cell migration, colony-forming potential) by interacting with and stabilizing misfolded mutp53. It should be noted that p53^R175H^ levels are not affected by HSC70 knockdown in CAL33 cells.	Stabilization of p53^R175H^ could be HSC70-independent, but the dependency on HSP70 needs to be clarified.	[23,55,64,65]
	p53^R158L^	DNAJA1/HDJ2 confers radio-resistance in SF763 cells.	UD	[67]
DNAJA3/Tid1	wtp53	Hypoxic and genotoxic stresses result in formation of a complex of DNAJA3/Tid1 and wtp53 through the J domain to promote p53 mitochondrial localization and transcription-independent apoptosis.	UD	[68,69]
DNAJB1/HDJ1	wtp53 (via MDM2)	The C-terminal region of DNAJB1/HDJ1 binds to, stabilizes, and inhibits MDM2, thus indirectly upregulating wtp53 activity.	Independent	[41]
	wtp53 (via PDCD5)	DNAJB1/HDJ1 inhibits wtp53’s apoptotic function through interaction with PDCD5 and contributes to cancer progression.	UD	[70]
	p53^R175H^	DNAJB1/HDJ1 interacts with conformational mutp53 (R175H), TAp73, and HSP70. This complex enhances chemo-resistance.	UD	[24]
	p53^R175H^	The interaction of DNAJB1/HDJ1 with p53^R175H^ is enhanced by chetomin, which restores the wtp53-like activity specifically in TOV-112D and CAL33 cells.	UD	[71]
DNAJB9/MDG1/ERdj4	wtp53	DNAJB9/MDG1/ERdj4 inhibits apoptosis under genotoxic stress by interacting with wtp53 through the J domain.	UD	[72]
	wtp53	The interaction of DNAJB9/MDG1/ERdj4 with p53 inhibits the H-RAS^V12^-induced senescence and promotes transformation in MEFs.	UD	[73]
	wtp53	Site-specific mutations in the *DNAJB9/MDG1/ERdj4* gene in JEG-3 cells reduce DNAJB9/MDG1/ERdj4 protein levels with increased wtp53 levels.	UD	[74]
DNAJC2/ZRF1	wtp53	DNAJC2/ZRF1 inhibits wtp53’s activity in human gastric cancer cell lines carrying wtp53, but not p53 mutations or p53 null.	UD	[75]
DNAJC7/TPR2	wtp53	DNAJC7/TPR2 inhibits colony-forming potential by stabilizing wtp53 in H1299 cells exogenously expressing wtp53.	UD	[76]

## Data Availability

Not applicable.
